# The photothermal effect of intense pulsed light and LipiFlow in eyelid related ocular surface diseases: Meibomian gland dysfunction, Demodex and blepharitis

**DOI:** 10.1016/j.heliyon.2024.e33852

**Published:** 2024-06-28

**Authors:** Hanqiao Li, Li Huang, Xie Fang, Zhiwen Xie, Xianwen Xiao, Shunrong Luo, Yuan Lin, Huping Wu

**Affiliations:** a Xiamen Eye Center and Eye Institute of Xiamen University, Xiamen, China; b Xiamen Clinical Research Center for Eye Diseases, Xiamen, Fujian, China; c Xiamen Key Laboratory of Ophthalmology, Xiamen, Fujian, China; d Fujian Key Laboratory of Corneal & Ocular Surface Diseases, Xiamen, Fujian, China; e Xiamen Key Laboratory of Corneal & Ocular Surface Diseases, Xiamen, Fujian, China; f Translational Medicine Institute of Xiamen Eye Center of Xiamen University, Xiamen, Fujian, China

**Keywords:** Intense pulsed light, LipiFlow, Meibomian gland dysfunction, Demodex, Blepharitis

## Abstract

The treatment and management of ocular surface diseases have shifted towards a co-treatment approach focusing on overall ocular surface homeostasis. When treating issues related to the eye, it is essential to not only focus on the damaged or disabled areas but also consider the larger picture. Meibomian gland dysfunction (MGD), Demodex infection, and blepharitis all interact at the eyelid site and can cause damage to the ocular surface to varying degrees. Palpebral lesions disrupt the balance of ocular surface homeostasis, leading to dry eye and keratitis. Traditional treatments, such as manual physical hot compress massage, have limited effectiveness due to the structure of the eyelid. However, intense pulsed light (IPL) technology uses penetrating light energy to generate heat energy, which can eliminate inflammation of capillaries or kill Demodex. Additionally, the LipiFlow thermal effect and physical compression provide a more vital and longer-lasting therapeutic effect on MGD by excluding other primary causes of ocular surface inflammation. Therefore, personalized treatment techniques based on photothermal effects may be effective. In the future, IPL and LipiFlow may potentially dismiss immune-inflammation factors causing ocular surface disease or block the delivery of systemic immune-related diseases.

## 1 Introduction

The eyelid is a crucial part of the ocular surface system, providing the first line of defense in protecting the eyes. Its role in maintaining the ecological balance of the ocular surface cannot be overstated. Meibomian glands, found in the upper and lower eyelids, produce oil that helps replenish the lipid layer of the tear film. When these glands malfunction, it can lead to a loss of lipid layer components, causing abnormal tear film distribution and instability, resulting in an imbalance of ocular surface homeostasis. Dry eye disease (DED) is a chronic and progressive ocular surface disease that causes dryness and a sensation of having a foreign object in the eye. Meibomian gland dysfunction(MGD) is a common cause of DED and leads to hyperevaporative dry eye due to abnormal lipid layer composition [1]. MGD can manifest in ways beyond gland absence or destruction. Demodex infestations and blepharitis-related keratoconjunctivitis can obstruct the meibomian gland opening and structural changes in the eyelid margin. In cases of eyelid Demodex infection, patients may show signs of corneal pathology alongside conjunctival inflammation [2]. When the eyelids are consistently irritated, the tissue and capillaries surrounding the eye surface are triggered to respond to inflammation. This can eventually worsen the symptoms of dry eyes. Bacterial lipase can impact the lipid layer of the tear film, changing its composition and leading to instability and inflammation of the ocular surface. If the meibomian ducts are blocked, bacterial growth increases, causing instability in the tear film due to more lipase production. This instability can damage the corneal epithelium and ultimately result in keratitis [3].

**Fig. 1:**
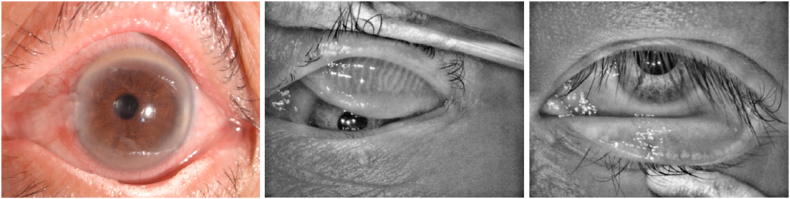
Meibomian gland dysfunction caused by the absence of the meibomian glands. Meibomian gland and ocular surface status influence each other, and meibomian gland dysfunction is one of the important factors of ocular surface damage.

One of the primary culprits behind evaporative dry eye disease is MGD. When the meibomian glands become obstructed, the tear film can destabilize, resulting in a shorter tear breakup time. This can cause superficial punctate keratopathy, which affects the lower cornea. Additionally, MGD can encourage bacterial growth in both the meibomian glands and eyelash area, leading to inflammation known as meibomitis. This inflammation can trigger ocular surface problems, including corneal cell infiltration, neovascularization, keratopathy, and conjunctivitis [4]. Demodex folliculitis can cause chronic anterior blepharitis, while Demodex brevis can cause posterior blepharitis, MGD, recurrent ptosis and refractory keratoconjunctivitis. Eyelash sampling, microscopic counting, and confocal microscopy in vivo are the main diagnostic methods for mites [5]. The roots of our eyelashes are home to Demodex, which feed on the follicles and epithelial cells of the glands found there. Unfortunately, this can physically damage the skin and cause epithelial hyperplasia and reactive hyperkeratosis. In addition, the mites lay eggs at the base of the eyelashes, which can cause the follicles to dilate and the eyelashes to misalign. Since Demodex lack excretory organs, they regurgitate undigested material that combines with epithelial cells, keratin, and eggs to form cylindrical deposits on the eyelashes [6]. These deposits contain proteases and lipases that can lead to irritating symptoms. The meibomian gland dysfunction caused by damaged meibomian gland is a vicious cycle for the ocular surface due to systemic conditions, infections, and the inflammatory state associated with the eyelid (Fig. 1).

Streptococcus and staphylococcus bacteria on mites' surfaces are directly linked to microbial blepharitis. They may also trigger an inflammatory response through toxins on their surface and inside the organism [7]. It has been observed that the mites that live on our skin carry bacteria known as Bacillus austeniae. These bacteria can activate the host's immune response. Even after the mite dies, it can still cause an inflammatory response by releasing some bacterial antigens, which trigger an inflammatory cascade in the host. This may be related to the dilation of capillary vessels at the palpebral margin and the vasodilation caused by inflammatory mediators [8]. Individuals who suffer from Demodex blepharitis may experience irritation in their eyelids due to lipolysis enzymes produced by the fat-fed parasite and an inflammatory response activated by the parasite's waste products. Identifying these initial symptoms is believed to result from the onset of blepharitis, which weakens the stability of the ocular surface and is caused by the Demodex [9].

Blepharitis is a condition that primarily affects the eyes and causes discomfort, discharge from the eyes, and redness in the conjunctiva. These symptoms can lead to light sensitivity and blurred vision. If left untreated, blepharitis can cause permanent changes in the shape of the eyelid and lead to visual defects such as keratopathy and corneal ulcers. The condition can be caused by inflammatory skin diseases, chronic infections at the border of the eyelid, or parasitic infections [10]. Blepharitis can impact the eyes and alter the appearance of the eyelids. It can also affect the meibomian gland, leading to meibomitis and MGD. This can result in blockage of the gland openings at the edge of the eyelids, which can impede proper secretion discharge. In addition, this condition can alter the components of the eyelids' oil layer, destabilizing the tear film and releasing inflammatory factors on the ocular surface [[11], [12], [13]]. The bacteria and their toxins, inflammatory factors, and free fatty acids on the ocular surface can further aggravate the inflammation of the meibomian glands and the palpebral margin [14,15]. Blepharitis impacts the eyelid margin and eyelashes. It is divided into two types, anterior blepharitis, and posterior blepharitis, with the former affecting the eyelid margin and eyelashes, while MGD characterizes the latter. To manage symptoms over the long term, experts recommend adhering to a daily eyelid cleaning routine and using therapeutic medications to reduce infection and inflammation [16]. To treat blepharitis effectively, it is important to address not only the condition itself but also any underlying MGD that may be contributing to the problem. Therefore, treating MGD is crucial for achieving a positive outcome in treating blepharitis and keratoconjunctivitis [17].

Due to the unique structure of the eyelids, traditional treatments such as hot compresses, palpebral margin care, and anti-inflammatory therapy may not effectively treat many patients [18]. Hot compress methods traditionally used to treat eye conditions apply heat to the outer surface of the eyelid. However, these methods are less effective because the heat must penetrate several layers of skin, muscles, and insulation of the eyelid before reaching the meibomian glands and their contents [19]. Light and heat effects are common for treating MGD and other ocular surface diseases related to the eyelid. When conventional drug therapies fail to manage such diseases effectively under the eyelid tissue, targeted treatment and management of the affected tissues through light and heat penetration can be a viable alternative with promising results [20]. Intense pulsed light (IPL) uses a specific spectrum of visible light to generate heat that can reduce the inflammatory response and prevent the growth of new capillaries. IPL treatment can be considered when other lifestyle changes, such as using lubricating eye drops or heat compress therapy, have failed to improve the signs and symptoms of dry eye [21]. DED is a multifactorial disease, and IPL treatment is safe and effective in reducing the signs and symptoms of dry eye associated with MGD, with the effects of IPL treatment lasting for several months [22]. LipiFlow is a well-established treatment for MGD. It is an automated procedure that simultaneously heats and evacuates the contents of the meibomian glands in both the upper and lower eyelids. With just one thermal pulse treatment, patients can experience safe and practical improvements in the subjective and objective parameters of MGD and dry eye [23]. A single heat pulse treatment produced long-lasting results, significantly improving meibomian gland function and reducing dry eye symptoms for over a year [24]. LipiFlow is currently regarded as a promising treatment for MGD as long as ocular surface inflammation has been ruled out. Based on this clinical approach, using light and heat therapy to target the meibomian gland could be a more efficient method to manage the inflammation associated with lipids in ocular surface diseases.

## 2 Mechanism of selective photothermal effect on the ocular surface

IPL is a broad-spectrum, high-intensity, incoherent light with wavelengths ranging from 515 nm to 1200 nm [25]. IPL is a treatment that uses photothermal and photochemical effects to act on skin tissue. It is commonly used to treat various skin conditions such as rosacea, acne, psoriasis, and lupus erythematosus. The technique also effectively treats pigmented lesions, bright red nevus, hemangioma, hidradenitis suppurativa, sarcoidosis of the skin, atopic dermatitis, and pigmented actinic lichen planus [26]. IPL has a wide wavelength range, which covers the absorption peaks of melanin, oxygen-containing and deoxyhemoglobin - the main chromophores found in human skin. This means that the thermal effect of hemoglobin absorption can promote the coagulation of abnormal eyelid telangiectasia, reducing the secretion of pro-inflammatory molecules [27]. According to recent research, it is believed that IPL treatment can help improve the function of the meibomian gland over a long period. This treatment can help alleviate symptoms by facilitating drainage of abnormal edema, dilating and clotting abnormal eyelid capillaries, interrupting the inflammatory cycle, and reducing the growth of bacteria and mites [28]. IPL, or intense pulsed light therapy, works by using selective photo pyrolysis to coagulate abnormal blood vessels near the eyelid, which helps to prevent the release of inflammatory markers and bacterial infection. IPL can also promote the secretion of the meibomian glands, which produce the oily layer of the tear film that helps to reduce evaporation and keep the eye lubricated [29]. The basic principle of IPL treatment is selective photopyrolysis. Mild side effects include tingling, swelling, erythema, blisters, scabs, and scattered lesion [30]. Reducing the pulse duration below the thermal relaxation time prevents non-specific thermal damage to the surrounding tissue.

LipiFlow is a treatment for MGD that involves applying a hot compress to the inner eyelid to dissolve the solid oil into liquid. This is combined with the pulse pressure method on the outer eyelid to effectively clear the oil and unclog the oil gland, improving MGD symptoms [31]. LipiFlow therapy involves the application of heat to both the upper and lower eyelid conjunctiva surfaces while simultaneously applying pressure on the external eyelid to express the meibomian gland. This therapy has been proven to effectively address the limitations of current treatments in clearing meibomian gland obstruction [32]. This new treatment method for obstructive meibomian gland dysfunction combines the benefits of heat therapy and physical expression. The meibomian glands are directly affected by applying heat and pressure to the eyelids. By touching the inner surface of the eyelid directly, heat transfer is reduced, leading to a significant improvement in efficacy. During treatment, the eyelids and meibomian glands are subject to heat and pressure simultaneously, which helps to dredge the meibomian glands during the heating process and minimize discomfort.

## 3 Combined treatment and management of eyelid-related ocular surface diseases

### 3.1 Therapeutic effect of meibomian gland dysfunction

IPL therapy is a safe and effective method for the treatment of evaporative DED caused by MGD and rosacea. After an average of four treatments, patients’ chronic symptoms were relieved. Additional treatment is usually required every six to twelve months to maintain symptom relief as an effective and safe treatment for evaporative DED [33]. IPL therapy can help improve both the overall symptoms and objective clinical indicators. There are several mechanisms through which IPL can achieve clinical improvement, including thrombosis of abnormal vessels under the skin around the eye, heating of meibomian glands and liquified membranes, activation of fibroblasts, and enhancement of the synthesis of new collagen fibers, eradication of Demodex and reduction of bacterial load in the eyelid, interference with the inflammatory cycle through the regulation of anti-inflammatory drugs and matrix metalloproteinases(MMPs), and reduction of the accumulation of skin epithelial cells and the risk of physical obstruction of the meibomian glands [34]. IPL treatment can help improve various symptoms in patients and related ocular surface indexes, meibomian gland function, macrostructure, and overall eyelid health status. In particular, IPL treatment can significantly improve the meibomian gland microstructure and effectively reduce inflammation in patients with MGD [35]. Studies have shown that three sessions of IPL therapy with meibomian gland expression (MGX) can significantly improve the objective presentation, subjective symptoms, meibomian gland function, and matrix metalloproteinase 9 (MMP-9) immunoassay results in patients with moderate to severe MGD. These results indicate that combining IPL and MGX is a safe and effective treatment for moderate and severe MGD. This therapy can also improve telangiectasia (visible blood vessels) of the eyelid margin, MMP-9 expression, and subjective symptoms [36,37].

Multiple mechanisms of action may be responsible for the effect of IPL on DED, although any of these mechanisms may explain it. As IPL is increasingly used for DED treatment, the specific role of these modes of action will become more apparent through further research. Studies have demonstrated that combining IPL and MGX can enhance tear film homeostasis and relieve ocular symptoms in patients with refractory MGD, with improvements observed between 6 and 32 weeks after initiating treatment [38]. Patients with DED have elevated levels of inflammatory molecules in their tears and the surface of their eyes. IPL treatment can interfere with the inflammatory cycle by either increasing the production of anti-inflammatory cytokines or decreasing the production of pro-inflammatory cytokines and tumor necrosis factor (TNF). Another benefit of IPL is its thrombogenic effect on the small blood vessels surrounding the meibomian glands and telomere dilating vessels in the eyelid and eyelid margin. This effect is thought to reduce the storage of inflammatory mediators, thereby eliminating the primary source of inflammation in the eyelids and meibomian glands. IPL can also decrease the volume density within the gland, causing it to transform from a viscous gel state in MGD to a liquid state, promoting its release [39]. Although LipiFlow has shown significant improvement in the apparent symptoms of MGD, it may have potential therapeutic benefits for both clinically observable objective symptoms and subjective impressions. However, long-term observation of MGD treated with LipiFlow reveals that dry eye symptoms persist, indicating that there may be other potential conditions causing dry eye symptoms in patients. The clinical population with dry eye symptoms is heterogeneous, and the tear film may be more susceptible to other adverse environmental conditions [40]. Recent studies have shown that single blepharitis-exclusion heat pulsation therapy is a highly effective long-term treatment for moderate to severe MGD. This treatment significantly reduces or eliminates the need for traditional dry eye therapies, such as daily use of artificial tears, hot compress therapy, or a combination of both [41]. Thermal pulsation therapy is a highly effective for restoring meibomian gland function and reducing dry eye symptoms. It can also improve other factors related to ocular surface health, such as tear break-up time (TBUT), lipid layer thickness (LLT), corneal fluorescence staining, and tear osmotic pressure. Lipiview's single 12-min vectoring heat pulse treatment has shown better results than a twice-daily hot compress combined with eyelid cleaning. Moreover, its therapeutic effect lasts 6–12 months [42]. Heat pulse treatment can be effective for patients with Sjogren's syndrome MGD suffering from dry eye symptoms. It can also help with obstructive meibomian gland dysfunction and low secretory meibomian gland dysfunction. However, the treatment's impact on obstructive meibomian gland dysfunction is more significant than its effect on low-secretory meibomian gland dysfunction [[43], [44], [45]]. It has been found that bilateral heat pulsation therapy is significantly better than taking oral doxycycline for three months. Since there are fewer risks associated with a single LipiFlow procedure than using doxycycline for a long time, using single heat pulsation therapy is a better option as an alternative to long-term antibiotic use [46].

### 3.2 Therapeutic effect of Demodex

**Fig. 2:**
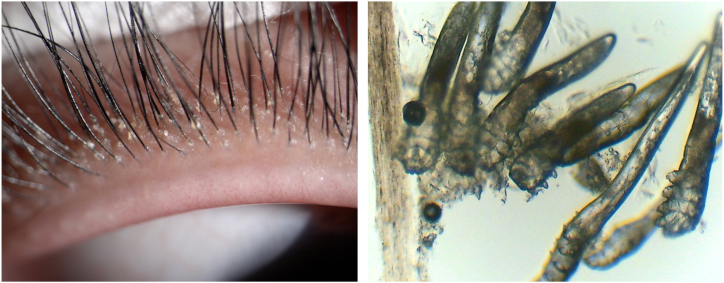
Demodex mite infection of the eyelid. The infection of mites destroys the eyelids and jointly causes inflammation of the ocular surface.

Demodex is a genus of small parasitic mites affecting mammals. The diagnosis of Demodex is mainly based on clinical evaluation and microscopic detection of Demodex in eyelashes. Symptoms such as blepharitis, blepharoconjunctivitis, ocular rosacea, eyelash disturbance, and conjunctival laxity may be suspected of vermicular infection [47]. A plausible mechanism of action for worm infection includes causing direct damage, acting as a carrier for bacteria, and inducing hypersensitivity. When Demodex infect the eyelid, it can cause a specific type of secretion to appear at the edge of the eyelid. The presence of Demodex can make the inflammation of the eyelid worse, leading to symptoms like burning and dryness. In cases where the Demodex infection affects the ocular surface, it can cause keratitis and damage to the corneal epithelium (Fig. 2).

Chronic and recurrent inflammation of the eyelid border can cause various malignant lesions of the eyelashes, including trichiasis, mascara disease, deformity, and cylindrical dander around the root of the eyelashes [48]. Applying IPL near the eyelid is an effective way to kill mites directly. Demodex organisms have chromophores, which makes them more sensitive to the energy delivered by IPL. The heat generated by IPL reaches the temperature required to destroy the mites and induces coagulation necrosis in the Demodex organism. Eradicating Demodex can indirectly reduce the bacterial load on the eyelid, reduce the immune response, and alleviate symptoms related to the eyelid margin and ocular surface [49]. IPL treatment can help to soften the secretion of meibomian glands and increase the excretion of palpebral sebum. A study using confocal microscopy showed that patients who underwent this treatment had decreased inflammatory cells and a significant increase in the length of corneal nerve fibers [50]. Demodex, which carry bacteria, can hurt the cells of the ocular surface, especially the meibomian gland cells. The toxic substances released by bacteria can increase the viscosity of palpebral sebum, thus causing further damage [51]. IPL treatment is an effective way to improve the inflammation of the meibomian glands. This is achieved by inhibiting the proliferation of Demodex, reducing the bacterial load of the eyelid and eliminating the source of inflammation. After the IPL treatment, most patients with Demodex blepharitis will receive meibomian gland massage. This helps excrete meibomian gland secretions more quickly than with massage alone [52].

### 3.3 Therapeutic effect of blepharitis

Blepharitis is an eye condition that causes inflammation of the edges of the eyelids. It leads to recurring symptoms such as itching, redness, tearing, and burning of the eye surface. Demodex blepharitis is a type of blepharitis that is chronic and recurrent, and it is often misdiagnosed. The most common symptom caused by inflammation is itching [53]. Patients with blepharitis have been found to have an increased number of precursor molecules that cause inflammation. This inflammation can trigger a cascade of events that leads to tear film instability. IPL therapy works by reducing chronic inflammation and improving the excretion of meibomian glands. This is achieved by reducing capillaries' dilation on the eyelids' margin [54]. It is more common for children to experience secondary conjunctiva and corneal problems after having blepharitis, known as blepharokeratoconjunctivitis (BKC). IPL mechanism treatment can rapidly improve the condition of BKC, shorten the duration of the disease, and reduce the need for hormone usage. It has a significant curative effect and can prevent the recurrence of blepharitis from the root cause [55]. The anti-inflammatory effect and heating mechanism of IPL may improve the ocular surface environment, including the eyelid, making it an effective adjuvant treatment for blepharitis-related diseases [56]. Detecting and treating blepharitis reduces clinical symptoms and prevents permanent structural damage. IPL treatment can be used along with meibomian gland dredging to control blepharitis and prevent the recurrence of keratoconjunctivitis. Clinical studies have shown that IPL can improve symptoms of moderate to severe acute blepharitis or blepharoconjunctivitis without causing any side effects. Measures such as the Ocular Surface Disease Index (OSDI) score, ocular surface staining, tear meniscus height, and non-invasive break-up time, as well as the Clinical Ocular Tolerance Score and meibomian gland expression, can be used to assess the morphological index of meibomian glands. Continuous IPL treatment can improve the clinical signs and symptoms of moderate to severe acute blepharitis or palpebral conjunctivitis and the morphology and secretion quality of meibomian glands [57].

## 4 Improvement and maintenance of ocular surface homeostasis by photothermal-assisted therapy

Animal studies have shown that IPL treatment can significantly reduce the mRNA levels of inflammatory factors such as tumor necrosis factor α (TNF-α), interleukin 17A (IL-17A), and interleukin 6 (IL-6). This reduction in inflammation is partly due to the inactivation of the transcription factor NF-κB pathway. IPL treatment can also prevent the excessive keratosis of meibomian glands, which breaks the cycle of pathological changes. Indirect IPL irradiation can also improve the biological function of the meibomian gland, which includes increasing the proliferation of meibomian gland basal cells and reducing oxidative stress. This suggests that indirect IPL irradiation can protect the distal tissue meibomian glands and reduce the development of MGD [58]. Inflammation is crucial in both the early and late stages of ocular surface disease. Tear film stability and osmotic pressure can be affected by various factors, leading to damage to the ocular surface, inflammation, and further damage. Cytokine and chemokine levels are linked to stimulation, tear film instability, tear production, and ocular surface integrity. IL-17A and IL-6 are involved in the development of DED caused by MGD. After IPL treatment, IPL can significantly reduce inflammatory markers of tear film instability in patients with DED caused by MGD, and the reduction of inflammatory factors correlates with improving some clinical symptoms and signs [59]. The therapeutic effect of IPL therapy may be derived from the regulation of inflammatory cytokines, including CXC motif chemokine ligand 1 (CXCL1), TNF-α, interferon γ (IFN-γ), CC motif chemokine ligand 11 (CCL11), interleukin 2 (IL-2), interleukin 6 (IL-6), and tissue inhibitor of metalloproteinase 1 (TIMP-1) [60]. IPL can disrupt the inflammatory cycle by either enhancing anti-inflammatory factors or inhibiting pro-inflammatory factors. It can reduce molecular factors in tears of dry eye patients, such as interleukin 1β (IL-1β) and interleukin 17F (IL-17F). IPL can also reduce the ratio of MMP9/TIMP1 and immune cells, including B cells [61]. These observations showed that the reduction of ocular surface inflammation confirmed the improvement in the indicators of ocular surface health.

IPL therapy is effective in reducing telangiectasia. This helps to prevent cytokine leakage through the facial arteries and orbital vessels, which are significant sources of inflammation in the meibomian glands. IPL therapy also helps downregulate proinflammatory cytokines, reducing ocular surface inflammation and improving signs and symptoms of MGD. This treatment helps enhance the integrity of the ocular surface, which can trigger a cascade of reactions, leading to improved corneal sensory nerve-ending activation, enhanced lacrimal nerve signaling, and increased tear secretion [62]. Clinically significant improvements in IPL treatment included tear break-up time (TBUT), corneal staining and eyelid margin measurements, measurement quality, meibomian gland excretion capacity, OSDI, and Standard Patient Evaluation of Eye Dryness Questionnaire (SPEED) [63]. IPL does not affect vision, eye pressure, lens opacity, fundus condition, or skin condition, and side effects are relatively rare but include discomfort, skin erythema, blisters, eyelash loss, and floaters, which are self-limiting [64]. IPL treatment not only enhances the structure of the meibomian glands but also improves their function by producing and secreting more lipids in the tear film of patients suffering from blepharitis. Additionally, younger patients can recover their meibomian gland function better when treated with IPL [65]. IPL treatment can improve symptom scores, ocular surface indexes, meibomian gland function, macrostructure, and eyelid health status. IPL treatment is particularly effective in improving meibomian gland microstructure and reducing inflammation in patients with MGD [66]. To summarize, deep cleaning of the eyelid border and IPL treatment can effectively reduce eye discomfort, relieve eyelid border inflammation, improve meibomian gland secretion, and reduce the number of Demodex on the eyelid border. This treatment is considered adequate for Demodex blepharitis. It's worth noting that Demodex are present in most people and only become harmful when they exceed a specific number, causing inflammation and tissue damage. Therefore, clinical treatment focuses on boosting the patient's immune system, controlling the mite population, and reducing inflammation and tissue damage [67]. Individuals with a Fitzpatrick skin type score greater than IV should not receive IPL treatment due to the increased risk of skin damage. Moreover, IPL treatment is primarily administered to the lower eyelid to prevent broad-spectrum light from penetrating intraocular tissues and causing potential harm [68].

It has been suggested that LipiFlow may reactivate the meibomian glandular structure, increasing the glands [69]. Thermopulsation therapy may significantly improve MGD by reducing CXCL chemokines in ocular surface tears of MGD patients, and the clinical therapeutic effect is good and well tolerated [70]. Treating MGD is an integral part of caring for patients with DED. Thermal pulsation is an effective treatment that significantly improves subjective and objective measurements of DED, including osmotic pressure of moderate and severe DED, MMP-9, TBUT, and OSDI. Treating meibomian glands with thermosensation can improve inflammation, and clinicians should be more aware of the diagnosis of MGD and have a lower threshold for treatment, especially for patients with elevated levels of MMP-9 and osmotic pressure [71]. TBUT and OSDI scores can be improved in patients after refractive surgery. The postoperative ratio of matrix metalloproteinase-9/tissue inhibitor matrix metalloproteinase-1 (MMP-9/TIMP1) was significantly higher than that of the preoperative matching level. Preoperative heat pulsation improved postoperative ocular surface signs and symptoms and reduced tear inflammatory factors, thus suggesting the rationality of the reduction of postoperative DED in patients with refractive surgery [72].

## 5 Management mode of ocular surface treatment combined with photothermal therapy

Some thermo-humid devices, eyelid massage, and cleaning devices have shown improvements in MGD. However, the treatment must be carried out regularly to maintain the therapeutic effect. Other instruments based on thermal pulsation and intense pulsed light technology have also demonstrated their ability to improve MGD status over extended periods [73]. Inflammation plays a crucial role in blepharitis and MGD-related DED. Chronic inflammation can cause structural changes in irregular blepharitis and mucosal junction replacement, which are not easy to reverse. Therefore, it is reasonable to expect that more than just eyelid cleaning will be necessary to observe any positive changes in eyelid rim irregularities and mucosal skin connections [74]. In individuals suffering from moderate to severe MGD, a combination of IPL therapy and MGX can help reduce the number and severity of symptoms and signs related to DED. Except for LLT, all test results showed significant improvement after a 15-week treatment period. These findings provide evidence for the effectiveness of IPL + MGX in mitigating the signs and symptoms of DED caused by MGD [75]. After receiving a series of IPL + MGX treatments, the patient reported experiencing an improvement in both their subjective symptoms and objective findings. This included better quality and quantity of marginal eyelid abnormalities and increased tear film stability and homeostasis. The potential mechanisms responsible for these improvements include the promotion of meibum melting, weakening abnormal blood vessels that release inflammatory factors, and the reduction of bacterial load at the eyelid rim [76].

**Fig. 3:**
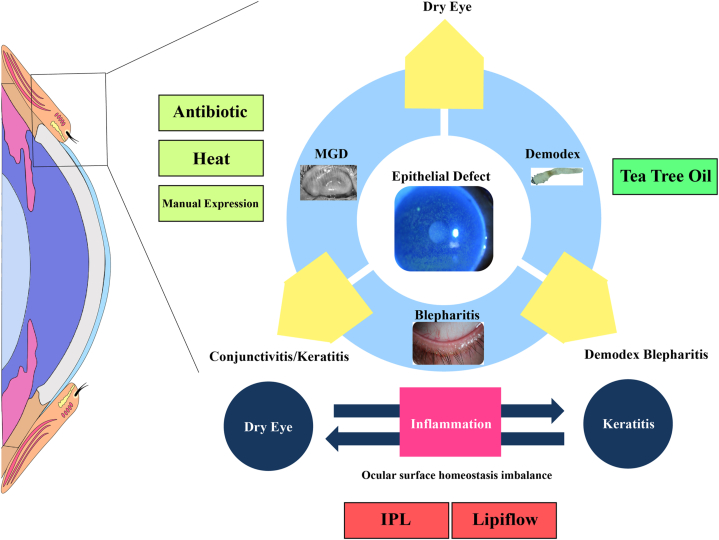
Treatment management mode of ocular surface homeostasis imbalance due to eyelid-related diseases. In addition to reducing damage to promote repair, eliminating inflammation is also an important part of current ocular surface management.

It is essential to manage the eye surface with a combination of treatments. When comparing treatments of the same duration, intense pulsed light in conjunction with sodium hyaluronate eye drops is more effective in improving clinical symptoms and signs, as well as killing mites, compared to using 5 % tea tree oil mite removal wipes along with sodium hyaluronate eye drops. Furthermore, this treatment method is safe, painless, and more accessible for patients to tolerate [77]. By using eyelid cleaning and tea tree oil cleaning wipes, along with three rounds of intense pulsed light treatment, it is possible to significantly decrease the number of Demodex, reduce scale, unblock meibomian gland opening, and alleviate blepharitis properties. This treatment can also improve symptoms of eye irritation [78]. Studies have explored the most practical combination of physiotherapy methods for BKC, including aerosol fumigation, meibomian gland massage, palpebral border cleaning, and cold compress [79]. Therefore, IPL combined therapy has a good effect on inflammation control and meibomian glands' functional recovery and can quickly restore the stable ocular surface microenvironment without significant adverse reactions, providing a new method for physical therapy of the ocular surface. During treatment, regular follow-up and treatment plan adjustment at any time are the key to personalized precision medicine. It's important to understand that eyelid-related conditions can cause an imbalance in the overall health of the eye. Dry eye and keratitis are dynamic conditions that are closely related to each other. Therefore, it's important to consider the health of the meibomian gland, eyelid tissue, and eyelid edge as a single unit. A comprehensive treatment plan that includes multiple management strategies is crucial for effective treatment. Previous methods such as hot compresses, massage, cleaning, and medication for the eyelids have been shown to be effective. However, we believe that IPL and LipiFlow technology can greatly improve the function of the eyelid glands, eliminate Demodex, and reduce inflammation on the eye's surface (Fig. 3).

In contrast, patients with significant eyelid inflammation and infection are unsuitable for LipiFlow. This new treatment, in which the meibomian glands are heated from the surface of the upper and lower eyelids while pressure is applied to express the meibomian glands, may not be suitable as an initial treatment for MGD in the form of overt inflammation or infection. Medication to treat inflammation and infection can be helpful in case of a blocked gland. Patients with hypersecretion disorders tend to experience significant improvement after blepharolipid treatment. Evacuating the meibomian glands catheter can help normalize the lacrimal lipid layer, leading to a more stable tear film [80]. Patients who have obstructed meibomian glands and respond positively to a particular treatment may require further treatment in the future, as the glands may become obstructed once again over time. Accurately predicting the optimal frequency of treatment for an individual is dependent on the duration of the treatment [81]. Both LipiFlow alone and meibomian gland massage have clinical effects on obstructive MGD. However, in patients treated with meibomian gland massage after LipiFlow, the treatment's efficacy and durability are stronger [82].

Although IPL and LipiFlow are relatively safe treatments and haves many benefits, we should also notice that they also have some shortcomings. According to the research, 14 % of the patients may have some side effects, including cheek swelling, hair loss, facial redness [83]. However, these effects usually disappear on its own within one week. After the treatment of LipiFlow, 5 % of the patients may complain about shortness temporary discomfort or pain [84]. In addition, LipiFlow is an expensive treatment, to some extent, that will limit its wide application. Therefore, when we choose the treatment for patients, we should have a thorough consideration in order to choose the most suitable treatment.

## 6 Conclusion

In the clinic, MGD is a common disease in the eyelid, which affects the inflammation of tear film and surrounding tissues. Parasitism of Demodex can destroy glands, produce secretions, and carry pathogenic microorganisms to make blepharitis more frequent and severe. When blepharitis continues with an inflammatory response, the ocular surface reflects progressive conjunctival disease. Considering the safety of IPL, its potential application in other hitherto unexplored ocular surface conditions should be investigated.

## Funding

This research was supported by grants from the Fujian Province Innovation and Entrepreneurship Talents (2021), Fujian Provincial Science Fund for Distinguished Young Scholars (2020D029), Fujian Provincial Fund for Middle-aged and Young Core Talents from Fujian Health Commission (2022GGB023), Bethune Charitable Foundation of Beijing (BJ-GY2021011J), Xiamen Municipal Guiding Project of Medical and Health (3502Z20214ZD1208, 3502Z20214ZD1209, 3502Z20214ZD1210, 3502Z20214D1211, 3502Z20224D1204), and Xiamen Municipal Guiding Project of Combination of Engineering with Medicine (3502Z20214ZD2193, 3502Z20214ZD2194, 3502Z20214ZD2195, and 3502Z20214ZD2196). The funding sources had no role in the design and conduct of the study: collection, analysis, and interpretation of the data; preparation, review, approval, and submission of the manuscript.

## Financial support

No conflicting relationship exists for any author.

## Availability of data and materials

Not applicable.

## Ethics approval and consent to participate

Not applicable.

## Consent for publication

Not applicable.

## Method of literature search

The databases and search engines used for this review was:PubMed. The search was conducted on the following date:November 15th, 2023. An overall search was conducted using the keywords: “intense pulsed light”, “Lipiflow”. In order to ensure that all studies published on the specific treatments discussed in the review were mentioned, individual searches were conducted for each treatment strategy using terms for the treatment in combination with the following keywords: meibomian glands, meibomian gland dysfunction, Demodex, and blepharitis. All years covered were included. The oldest included article was from 1977. No further articles meeting the inclusion criteria were found cited in the reference list of the included articles or through other sources. All results were evaluated through first examining title and then abstract for relevance to the subject and checking against exclusion criteria. Exclusion criteria were case reports which did not contribute new information and studies only described as abstracts.

## Data availability statement

NO. Data will be made available on request.

## CRediT authorship contribution statement

**Hanqiao Li:** Writing – original draft, Investigation. **Li Huang:** Writing – original draft, Methodology, Investigation. **Xie Fang:** Methodology, Investigation. **Zhiwen Xie:** Methodology, Investigation. **Xianwen Xiao:** Methodology, Investigation. **Shunrong Luo:** Methodology, Investigation. **Yuan Lin:** Writing – review & editing, Visualization, Conceptualization. **Huping Wu:** Writing – review & editing, Project administration, Conceptualization.

## Declaration of competing interest

The authors declare that they have no known competing financial interests or personal relationships that could have appeared to influence the work reported in this paper.
